# Seasonal Dynamics of Benthic Macroinvertebrate Community Assembly in a Subtropical Monsoon‐Driven Mountain Stream

**DOI:** 10.1002/ece3.72109

**Published:** 2025-09-30

**Authors:** Yihao Ge, Yan Xue, Ke Chen, Zhiwen Gan, Ke Xu, Changrui Zhao, Yuzhou Zhang, Yunzhi Yan

**Affiliations:** ^1^ Collaborative Innovation Center of Recovery and Reconstruction of Degraded Ecosystem in Wanjiang Basin Co‐Founded by Anhui Province and Ministry of Education, and School of Ecology and Environment, Anhui Normal University Wuhu China; ^2^ The Key Laboratory of Aquatic Biodiversity and Conservation, Institute of Hydrobiology Chinese Academy of Sciences Wuhan China

**Keywords:** benthic macroinvertebrates, community assembly, dispersal dynamics, environmental filtering, metacommunity structure

## Abstract

Seasonal variation in community assembly influences biodiversity patterns, yet its dynamics under monsoon‐driven hydrology remain underexplored in subtropical mountain streams. We applied the Elements of Metacommunity Structure (EMS) framework and variation partitioning to assess seasonal shifts in benthic macroinvertebrate communities across dry, normal, and wet phases in a subtropical monsoon‐driven mountain stream. Assembly patterns transitioned from Quasi‐Clementsian (dry season) to Clementsian (normal flow) and Nested structure (wet season). Deterministic environmental filtering—mediated by water temperature, dissolved oxygen, conductivity, water depth, and flow velocity—dominated during dry and intermediate phases. Conversely, stochastic dispersal (mass effects) prevailed during high‐flow monsoon periods, driven by enhanced connectivity. By quantifying the hierarchical balance between deterministic and stochastic drivers, our study advances understanding of how hydrological seasonality structures metacommunities. These findings underscore the importance of integrating intra‐annual hydrological variability into stream biodiversity assessments and management frameworks.

## Graphical abstract

This study examines how seasonal hydrological variations influence macroinvertebrate community assembly in the Huishui River using the EMS framework and variation partitioning analysis. The findings reveal that dispersal processes dominate during high‐flow monsoon periods, environmental filtering prevails in drought phases, and both processes contribute during transitional periods, highlighting the importance of intra‐annual hydrological dynamics for river bioassessment and management.

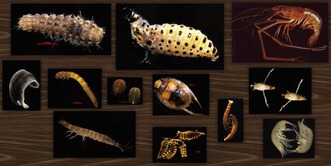

## 1 Introduction

A central goal in community ecology is to identify temporal patterns in species distributions and abundance dynamics, while elucidating the mechanisms driving community assembly (Ontiveros et al. 2021; Jia et al. 2024; Liu et al. 2024). Understanding these assembly mechanisms provides critical insights into Earth's intricate web of life, serving as an essential foundation for biodiversity conservation, ecosystem function maintenance, ecological restoration practices, and evidence‐based environmental management (Huang et al. 2018; Li et al. 2025). Contemporary metacommunity frameworks propose that community assembly mechanisms operate on a continuum between deterministic and stochastic processes (Csercsa et al. 2019; Chiu et al. 2020). The classic niche theory posits that deterministic processes—such as environmental filtering and biotic interactions—drive niche differentiation among coexisting species and are considered the primary forces in community assembly (Poff 1997; Chase and Myers 2011; García‐Girón et al. 2020). In contrast, stochastic processes, rooted in neutral theory, include random dispersal, extinction, speciation, and ecological drift, which are governed by chance ecological events (Hubbell 2001; Tonkin et al. 2018; Liu et al. 2021). Modern theoretical frameworks emphasize the need to integrate both deterministic and stochastic processes to fully explain community assembly mechanisms (Leibold and Mcpeek 2006; Adler et al. 2007; Garcia‐Giron et al. 2019).

Modern empirical studies demonstrate that community assembly is strongly influenced by ecological context, with the relative importance of deterministic and stochastic processes varying significantly across temporal scales (Heino et al. 2012; Liu et al. 2024). However, while numerous studies have examined context dependency in community assembly, relatively few have focused on how the relative contribution of stochastic versus deterministic processes changes systematically over time, particularly in lotic systems shaped by monsoonal hydrology. In such systems, temporal variation in hydrological connectivity modulates the strength and dominance of different assembly processes rather than time itself being a source of stochasticity (Fernandes et al. 2014; Li et al. 2021). Temporal dynamics in both environmental conditions and the spatial configuration of natural habitats can drive shifts in community structure and the underlying assembly mechanisms over time (Fitzgerald et al. 2017; Liu et al. 2024). This effect is particularly pronounced in lotic systems subject to monsoonal pulses. Dynamic flow regimes in these running‐water habitats result in substantial inter‐seasonal hydrological shifts, which are synchronized with predictable oscillations in physicochemical conditions (e.g., water quality and nutrient availability) and metacommunity linkages (Leung and Dudgeon 2011; Li et al. 2020; Chi et al. 2024). These periodic fluctuations can drive temporal variation in community structure and its underlying drivers (Fitzgerald et al. 2017; Li et al. 2020). Elevated hydrological fluxes promote dendritic network connectivity in lotic ecosystems (Thomaz et al. 2007). This coupling reduces habitat environmental heterogeneity while enabling cross‐tributary dispersal through expanded hydrological corridors (Bozelli et al. 2015). Consequently, spatial processes driven by mass effects—where community dynamics are largely shaped by intense dispersal, allowing species to persist in suboptimal habitats via continuous immigration from source areas—are expected to significantly influence community assembly during the monsoon season (Leibold et al. 2004; Hill et al. 2017). In contrast, low‐flow conditions during the dry season, driven by precipitation deficits, lead to stream network contraction. This hydrological regime increases spatial heterogeneity through enhanced environmental gradients while simultaneously creating biogeographical barriers to aquatic dispersal (Thomaz et al. 2007; Dray et al. 2012). These spatiotemporal constraints amplify environmental filtering pressures and restrict metacommunity connectivity across the dendritic riverine landscape (Tonkin et al. 2018). Therefore, seasonal dynamics in stream systems may mediate critical transitions in assembly rules. Despite this theoretical framework, empirical studies capturing such seasonal transitions in metacommunity organization remain limited, especially in subtropical mountain streams, where strong altitudinal gradients and regional endemism may further shape the balance of assembly mechanisms. Incorporating seasonal variability into empirical research enables river ecologists to gain a more comprehensive understanding of community assembly processes. In contrast, most existing studies rely on static or single‐time‐point sampling—an approach that often fails to capture the full extent of seasonal dynamics and temporal shifts in assembly mechanisms (Fernandes et al. 2014; Liu et al. 2024).

Aquatic macroinvertebrates are an exemplary group of organisms for evaluating the impact of hydrological fluctuations driven by monsoon variations on community assembly mechanisms, owing to several key characteristics (Li et al. 2020; Jiang et al. 2021). First, macroinvertebrates are generally sensitive to fluctuations in hydrological and environmental conditions, with many taxa exhibiting marked responses to seasonal changes. Under intense environmental stress, some species may migrate to more favorable habitats, while others may experience population declines. This ecological sensitivity makes macroinvertebrates widely used as bioindicators for assessing seasonal variation in aquatic ecosystems (Heino et al. 2015; Liu et al. 2024). Second, macroinvertebrates exhibit a diverse range of dispersal‐related functional traits, including body size (e.g., small‐sized ≤ 9 mm, medium‐sized 9–16 mm, and large‐sized > 16 mm), swimming capabilities, and dispersal pathways (Heino 2013). They utilize two primary dispersal vectors: aquatic propagules disperse mainly through passive drift or localized crawling/swimming, while semiaquatic taxa rely on aerial dispersal during terrestrial life stages, which may include compensatory upstream flights via powered flight or wind‐assisted passive transport (Tonkin et al. 2018; Ge et al. 2023). These dispersal mechanisms are significantly influenced by periodic monsoon oscillations, which can alter both community structure and the functional composition of dispersal‐related traits, thereby modulating the relative importance of spatial processes in community assembly dynamics (Tonkin et al. 2018). Finally, the morphological and phenological characteristics of macroinvertebrates exhibit pronounced seasonal variation, which plays a crucial role in shaping the temporal dynamics of community assembly. For example, caddisflies (Trichoptera) often have univoltine life cycles, with aquatic larvae developing through spring and summer, then emerging as winged adults in late summer or early autumn. These adults are short‐lived (often only a few days to weeks), leading to pronounced seasonal peaks in abundance and turnover in community composition between seasons (Morse et al. 1984). These seasonal variations in trait expression influence both environmental filtering and spatial structuring processes (Ge et al. 2023). Among macroinvertebrates, aquatic insects represent a dominant and functionally important subgroup in stream ecosystems. Unlike organisms with relatively stable life‐history strategies and continuous aquatic residency—such as fish and mollusks, which often display consistent community patterns over time due to passive dispersal along stream corridors—aquatic insects exhibit complex metamorphic life cycles (Li et al. 2016; Tonkin et al. 2018). These insects develop as wingless aquatic larvae and emerge as winged terrestrial adults, introducing clear temporal discontinuities in habitat use. The marked seasonal variation in their morphological and phenological traits introduces a high degree of temporal turnover in community composition and modulates the balance between deterministic and stochastic assembly mechanisms. This phenological decoupling between aquatic and terrestrial stages adds a distinct layer of temporal and spatial complexity to their metacommunity dynamics (Bilton et al. 2001; Heino et al. 2015; Tonkin et al. 2018). The phenological decoupling between aquatic and terrestrial phases adds a layer of spatial and temporal complexity to their community assembly processes (Liu et al. 2024). Notably, empirical studies of macroinvertebrate metacommunities in the lotic systems of the East Asian monsoon region remain scarce. Critical knowledge gaps persist in mechanistically linking monsoon‐mediated hydrological regimes to the seasonally shifting assembly mechanisms in these systems (Li et al. 2020; Liu et al. 2024).

This study integrates statistical modeling with the Elements of Metacommunity Structure (EMS) framework to examine temporal variations in the assembly mechanisms that govern benthic macroinvertebrate communities in the subtropical monsoon‐regulated Huishui River, China. We systematically assessed the relative contributions of environmental filtering and spatial processes across three hydrologically distinct seasons: dry, intermediate, and wet. Our central hypothesis posits that monsoon‐driven hydrological dynamics mediate both community composition and assembly mechanisms. Based on the documented interactions between life‐history strategies, hydrological regimes, and local habitat conditions, we predicted significant seasonal shifts in the key ecological factors driving the community assembly of stream macroinvertebrates. Specifically, we proposed four mechanistic hypotheses: (1) Environmental filtering dominance in the dry season: During the dry season, as river networks become increasingly fragmented, hydrological isolation enhances environmental heterogeneity and weakens spatial structuring capacity. These changes promote species sorting via environmental filtering, leading to community divergence along environmental gradients and resulting in spatially structured, gradient‐aligned patterns (e.g., Clementsian or Gleasonian). (2) Spatial process dominance during monsoons: During the monsoon season, community assembly is primarily driven by spatial processes due to enhanced hydrological connectivity. Hydrologically mediated mass effects and source‐sink dynamics override environmental filtering and lead to spatial homogenization. (3) Flood‐induced nested metacommunity architecture: Monsoonal flood regimes would induce a nested community structure, where some species are lost due to recurrent flood pulses, resulting in a series of nested subsets of progressively larger assemblages.

## 2 Materials and Methods

### 2.1 Study Area

**FIGURE 1:**
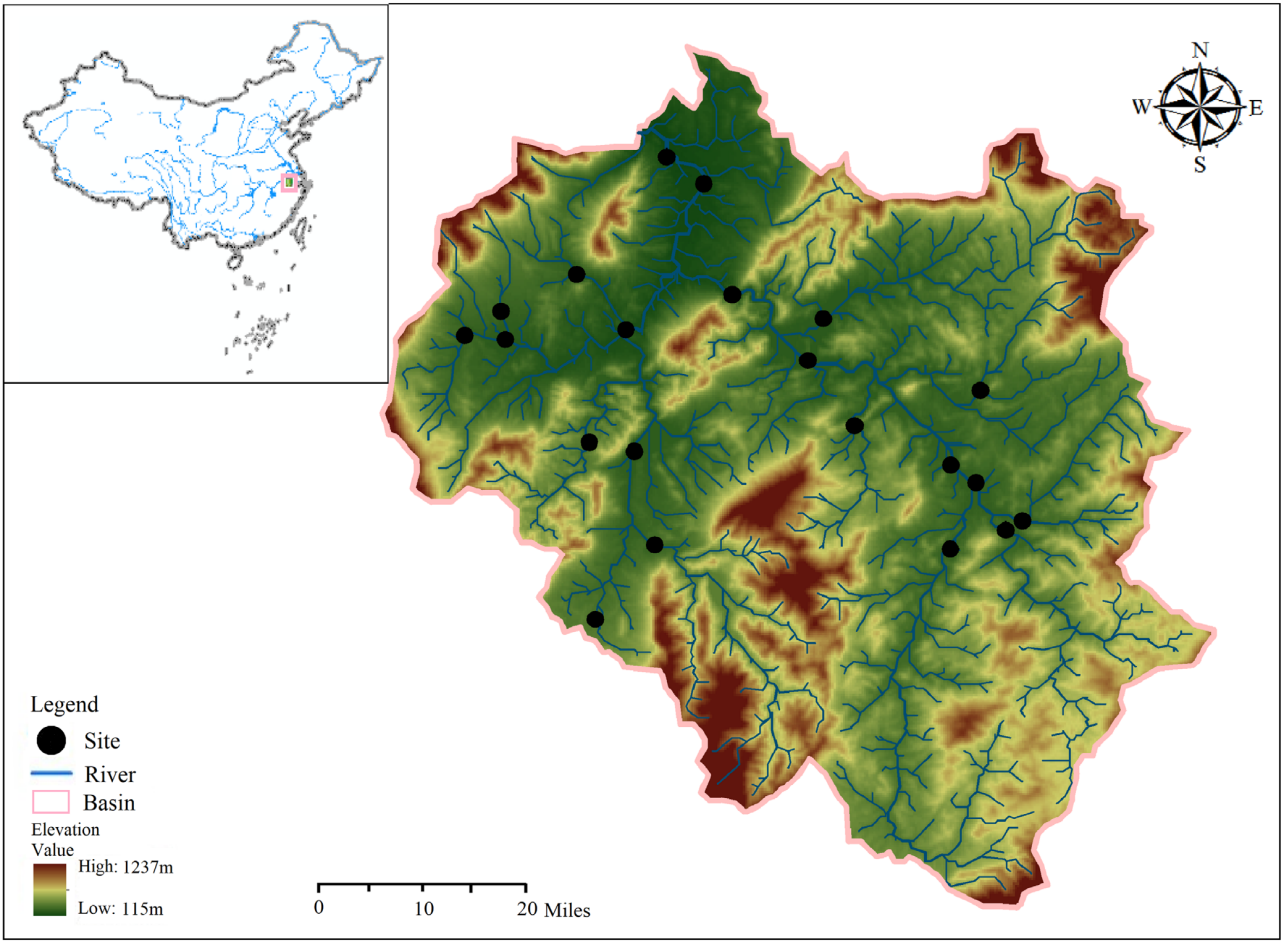
Location of the Huishui River Basin in China, and the distribution of the 21 stream sites in the study area.

This study was conducted in the Huishui River (30°14′–30°25′ N, 118°20′–118°33′ E), the primary tributary of the Qingyi River within the Yangtze River basin, China. The river drains a substantial area, with a main channel length of approximately 119 km, a basin area of around 1083 km^2^, and an average slope of 1% (Figure 1). The study area lies at the biogeographic ecotone between the Palaearctic and Oriental realms. The mean annual temperature is approximately 15.5°C, with average annual rainfall of 1521.5 mm. Rainfall in the Huishui River basin varies considerably across seasons. The majority of precipitation occurs during the wet season (June to August), accounting for 40% of the annual total. In contrast, only about 17.17% of precipitation falls during the dry season (from November to February), with the remaining months (March to June and September to October) characterized as normal seasons (Hao et al. 2024). As a secondary tributary of the Yangtze River, the Huishui River flows through the ecotone between the Palaearctic and Oriental regions. The study area features a diverse and complex topography, with elevations ranging from 115 to 1237 m above sea level. This varied topography supports exceptionally high biodiversity and is home to a significant number of relict species (Qiao et al. 2023). Given the rich diversity of rare and endemic fish species in the Huishui River, the Chinese Ministry of Agriculture established the National Aquatic Germplasm Reserve of Endemic Fish in the river in 2009.

### 2.2 Field Survey

Benthic macroinvertebrates were collected from 21 stream sites during three distinct seasons in 2021: normal (May), wet (August), and dry (December). The sampling network consisted of nine sites along the mainstem (including the Hui River and the main river channel) and twelve additional sites located in tributary streams, with one site selected per tributary. This design aimed to capture the spatial heterogeneity of the stream network while maintaining hydrological consistency across the sampling area. At each site, samples were obtained from representative microhabitats (e.g., riffles, runs, and pools) using a Surber sampler (30 × 30 cm, with 500 μm in mesh size). In each dominant habitat type, three replicate samples were taken by disturbing the substrate upstream of the sampler for a fixed duration to dislodge organisms. The collected material was immediately preserved in 70% ethanol in the field and transported to the laboratory for further processing (Barbour 1999). All specimens were subsequently identified to the lowest possible taxonomic level (species or genus) in the laboratory, using relevant taxonomic references (e.g., Morse et al. 1984; Dudgeon 1999; Epler 2001; Zhou et al. 2003).

### 2.3 Environmental Variables

During the investigation, altitude, latitude, and longitude were recorded using a portable GPS (UniStrong MG721W). At each sampling site, 1 L of surface water was collected and immediately stored at −4°C in a portable refrigerator. To ensure the stability of the samples during transportation, water samples were kept at low temperatures, protected from light, and transported to the laboratory for the analysis of environmental variables, including total nitrogen (TN), total phosphorus (TP), chemical oxygen demand (COD), and ammonia nitrogen (NH_4_^+^‐N). Preservation methods were applied based on the characteristics of each variable. For instance, to prevent microbial activity and chemical transformations, samples for TN and TP analysis were acidified with sulfuric acid (H_2_SO_4_) to a pH ≤ 2. These procedures were implemented in accordance with standard protocols to maintain the integrity and accuracy of the measurements. A portable multi‐parameter water quality analyzer (Hach HQ40D) was used to record water temperature (WT), transparency (Trans), pH, dissolved oxygen concentration (DO), dissolved oxygen saturation (DOS), electrical conductivity (EC), total dissolved solids (TDS) and other physical and chemical indicators of water. Current velocity (CV)was measured at five random locations at each site with a portable flowmeter (FP11, USA). Water depth was measured using a graduated rod, with five measurements taken across a transect at each site and averaged to obtain site‐level values. Land cover within the riparian zone (within a 100 m buffer) was classified using high‐resolution satellite imagery (e.g., Sentinel‐2) and analyzed using ArcGIS 10.6. Land use types were visually interpreted and categorized as forest, agriculture, urban, or other, following standard land‐use classification criteria. Benthic substrate was classified into five types based on particle size: sand and silt (< 2 mm); gravel (2 mm ≤ size < 32 mm); pebbles (32 mm ≤ size < 64 mm); cobbles (64 mm≤size < 256 mm); and boulders (≥ 256 mm). The percentage of each substrate type was visually estimated at each site using a 1 m^2^ grid (Kondolf 1997).

### 2.4 Spatial Factors

Moran Eigenvector Maps (MEM) were used to model the spatial relationships of community structure among sites, thereby creating spatial variables. MEM, formerly known as Principal Coordinates of Neighbor Matrices (PCNM; Borcard and Legendre 2002), was applied to identify and model spatial structures at various scales, from large‐scale (the entire sampling area) to small‐scale (distances among sampling sites). MEM can model any type of spatial structure, providing an appropriate spatial framework for realistic geographic sampling areas. PCNM was used as a spatial variable to analyze spatial variation in community structure at different spatial scales. From all the PCNMs, we retained those associated with significant Moran's I and positive eigenvalues for further analysis, as they represent positive spatial autocorrelation (Gilbert and Bennett 2010). The spatial analysis was conducted using the “pcnm” function from the vegan package in R v3.6.1 (R Core Team 2018), resulting in a total of 15 PCNM axes (PCNM1‐PCNM15) with positive eigenvalues.

### 2.5 Data Analysis

#### 2.5.1 Environmental Characteristics

A one‐way, repeated‐measures analysis of variance (ANOVA) was initially employed to detect differences in environmental factors (log‐transformed to improve normality) across the seasons. Fluctuations in hydrological connectivity can affect between‐site environmental heterogeneity, potentially confounding the examination of the effects of connectivity on community assembly mechanisms (i.e., environmental vs. spatial factors) across different periods. Therefore, we adopted the Permutational Analysis of Multivariate Dispersion (PERMDISP; Legendre and Legendre 2012) to assess environmental heterogeneity for each season. This analysis examines the differences in the average distance of sampling sites to the group centroid in multivariate space, with a higher average distance indicating greater environmental heterogeneity (Anderson 2006). PERMDISP was performed based on Euclidean distances, considering all standardized environmental parameters (except for pH).

#### 2.5.2 Species Composition

We performed nonmetric multidimensional scaling (NMDS) using the Bray–Curtis similarity distance based on abundance data to distinguish differences in community structure across seasons (Legendre and Legendre 2012). Similarity percentage analysis (SIMPER) was then used to identify species that contributed most to community dissimilarity among seasons (Clarke 1993). Additionally, one‐way analysis of similarities (ANOSIM) with 999 permutations was employed to assess whether seasonal differences in macroinvertebrate community composition were significant (Clarke 1993). ANOVAs were conducted using SPSS statistical software (version 22.0), while PERMDISP, ANOSIM, and SIMPER analyses were performed with PERMANOVA+ for PRIMER (Anderson 2006). NMDS was conducted in the R environment using the vegan package (R Core Development Team 2018).

#### 2.5.3 Metacommunity Structures

To assess seasonal patterns in benthic species distribution across sites, we applied the Elements of Metacommunity Structure (EMS) framework (Leibold and Mikkelson 2002; Presley et al. 2010; Henriques‐Silva et al. 2013). Using a site‐by‐species incidence matrix, we evaluated three key metrics: coherence, species turnover, and boundary clumping. The matrix was first ordered by reciprocal averaging (correspondence analysis), which represents a latent environmental gradient. Coherence was assessed by comparing observed embedded absences to a null model to detect overall structure. Significant coherence led to further evaluation of species turnover (via *z*‐scores) and boundary clumping (using Morisita's index and chi‐square tests) to distinguish among idealized metacommunity types—such as Clementsian, Gleasonian, evenly spaced, nested, or checkerboard structures. All analyses were conducted using the “Metacommunity” function in the *metacom* R package (Dallas 2014). This framework helps determine whether species distributions are structured by gradients, nestedness, or competitive exclusion, providing ecological insights into the assembly processes shaping the benthic communities.

#### 2.5.4 Variation Partitioning

Distance‐based redundancy analysis (db‐RDA; Legendre and Anderson 1999), based on the Bray‐Curtis resemblance matrix, was used to identify key environmental and/or spatial variables influencing macroinvertebrate community structure. db‐RDA was performed using the “ordiR2step” function in the vegan R package to conduct forward selection for both environmental and spatial (MEM) variables, with significance determined by *p* < 0.05 after 999 random permutations (Oksanen et al. 2016). The goal was to examine the relative contributions of local environmental variables and spatial variables (MEM eigenvectors) to the patterns of metacommunity structure in river macroinvertebrates.

Variance partitioning analysis (Legendre and Legendre 2012) was then conducted using the “varpart” function in vegan to partition the total variation in macroinvertebrate metacommunity structure into unique and shared contributions of environmental and spatial predictors. The total variation was assessed through constrained RDA ordinations, and adjusted *R* ^2^ values were reported for the pure and shared contributions of spatial and environmental variables. Statistical significance of the unique and shared contributions of each predictor set was evaluated using the “anova” function in the vegan R package (Oksanen et al. 2016).

## 3 Results

### 3.1 Environmental Characteristics and Species Composition

Nearly half of the 15 analyzed environmental parameters exhibited significant seasonal differences (*p* < 0.05). Specifically, physical properties (e.g., dissolved oxygen (DO), water temperature (WT), electrical conductivity (EC), water depth (WD), and current velocity (CV)) showed substantial seasonal fluctuations, while chemical characteristics (excluding pH) remained relatively stable over time (Table 1). PERMDISP analysis revealed no significant difference in environmental heterogeneity among seasons (*F* = 1.507; *p* = 0.229). However, in terms of environmental variation, the wet season exhibited greater heterogeneity compared to the dry and normal seasons (Figure S1).

**TABLE 1:** Results of One‐way, repeated‐measures analysis of variance (ANOVA), also showing the mean value (Mean) and standard deviation (SD) of environmental variables at the three sampling periods in Huishui River.

Environmental variables	Abbreviation	Normal		Wet		Dry		*F*	*p*
		Mean	SD	Mean	SD	Mean	SD		
River width (m)	RW	26.31	25.97	34.93	42.15	22.71	29.18	0.75	0.480
Water depth (cm)	WD	31.31	14.49	49.94	22.08	36.6	19.76	4.23	**0.017**
Current velocity (m/s)	CV	0.22	0.09	0.38	0.28	0.17	0.08	7.99	**0.001**
Water temperature (°C)	WT	20.1	1.44	32.56	3.05	8.85	2.94	441.7	**0.000**
Land Cover (%)	LC	0.30	0.30	0.27	0.29	0.26	0.29	0.12	0.903
Percentage of boulder (%)	%Boulde	0.21	0.22	0.32	0.24	0.2	0.22	1.76	0.180
Percentage of cobble (%)	%Cobble	0.31	0.23	0.20	0.18	0.32	0.26	1.85	0.166
Percentage of pebble (%)	%Pebble	0.21	0.17	0.14	0.13	0.2	0.19	1.03	0.363
Percentage of gravel (%)	%Gravel	0.05	0.06	0.18	0.28	0.05	0.05	4.22	**0.019**
Percentage of sand and silt (%)	%Sand and Silt	0.22	0.27	0.1	0.30	0.22	0.27	0.39	0.678
pH	pH	7.55	0.36	7.91	0.64	8.21	0.45	65.63	**0.000**
Dissolved oxygen (mg/L)	DO	9.18	0.91	5.21	1.64	4.96	0.89	82.39	**0.000**
Conductivity (μs/cm)	EC	152.59	43.34	232.69	93.3	165.64	51.81	8.77	**0.000**
Total phosphorus (mg/L)	TP	0.03	0.02	0.1	0.15	0.05	0.05	1.50	0.254
Total nitrogen (mg/L)	TN	2.37	0.93	1.99	0.67	2.69	0.99	2.51	0.246

*Note:* Bold values indicate statistically significance at *p* < 0.05.

**FIGURE 2:**
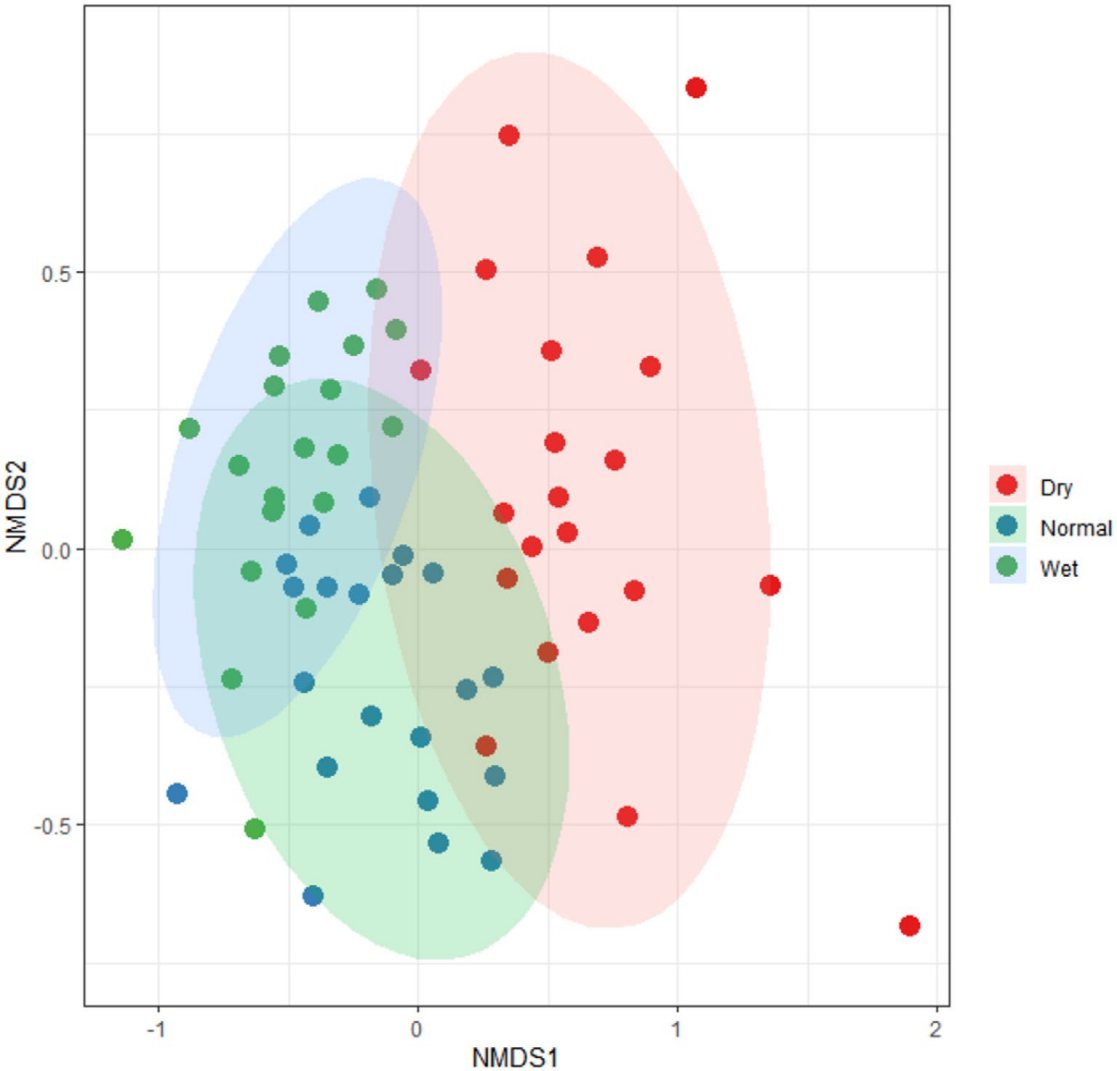
Non‐metric Multidimensional Scaling (NMDS) ordination of macroinvertebrate communities in the Huishui River during Dry, Normal, and Wet seasons based on Bray‐Curtis dissimilarities. Stress = 0.18, indicating a moderate representation of the community dissimilarity structure.

A total of 139 macroinvertebrate taxa were identified, spanning eight classes, 20 orders, and 71 families. The highest number of taxa (85) and individuals (6296) were detected during the normal season, compared to the dry season (82 taxa, 3024 individuals) and wet season (77 taxa, 1652 individuals) (Table S1). NMDS analysis revealed significant differences in community composition among seasons (Figure 2). ANOSIM analysis confirmed that all pairwise comparisons of communities across seasons were significantly different (Global *R* = 0.45, *p* = 0.001).

SIMPER analysis indicated that during the wet season, the community was predominantly composed of *Cryptochironomus* sp., *Baetis* sp., *Radix swinhoei*, *Hydropsyche* sp., *Caridina* sp., and *Heptagenia* sp., which together accounted for 40.30% of within‐group similarity. The dry season was dominated by *Cryptochironomus* sp., *Corbicula fluminea* , *Baetis* sp., *Trissopelopia* sp., and *Heptagenia* sp., contributing 50.10% to within‐group similarity. Finally, the normal season saw dominance from *Stictochironomus* sp., *Turbellaria* sp., *Corbicula fluminea* , *Cryptochironomus* sp., *Polypedilum* sp., *Radix swinhoei*, and *Baetis* sp., explaining 42.60% of within‐group similarity (Table S2).

### 3.2 Seasonal Variation in Idealized Metacommunity Structures

The first step of the EMS analysis revealed significantly negative coherence (*z*‐score < 0) in all three seasons, suggesting that species generally responded to the same environmental gradient. However, species turnover varied with season. In the wet season, turnover was positive but not significant (*z* = 0.27), corresponding to a clumped species loss structure. In the normal season, turnover was significantly positive (*z* = 3.10, *p* < 0.01), consistent with a Clementsian gradient. In the dry season, turnover was positive but statistically non‐significant, reflecting a quasi‐gradient structure (Table 2; Figure S1). Additionally, the metacommunities in all seasons exhibited significantly positive boundary clumping (Morisita's index > 1), indicating that changes in community structure were primarily driven by differences in taxa groups across sites. The idealized metacommunity structures that best fit the observed patterns in each season were: clumped species loss (wet season), quasi‐Clementsian gradients (dry season), and Clementsian gradients (normal season) (Table 2).

**TABLE 2:** Results of analyses of coherence, species turnover, and boundary clumping for macroinvertebrate metacommunities in the wet, normal, and dry seasons. The best‐fitting metacommunity structures are also determined.

Season	Coherence			Species turnover			Boundary clumping	
	Observed absences	Expected absences	*z*‐score	Observed replacements	Expected replacements	*z*‐score	Morisita's index	Best‐fitting structures
Normal	751	1355.91	−24.83***	23,993	19172.51	3.10**	1.35***	Clementsian
Wet	1707	3193.43	−19.91***	15,153	14583.78	0.27	1.81***	Clumped species loss
Dry	738	1272.81	−18.37***	25,462	22423.08	1.63	1.44***	Quasi‐Clementsian

*Note:* **p < 0.01 and * * *p < 0.001.

### 3.3 Seasonal Variation in Driving Forces

**FIGURE 3:**
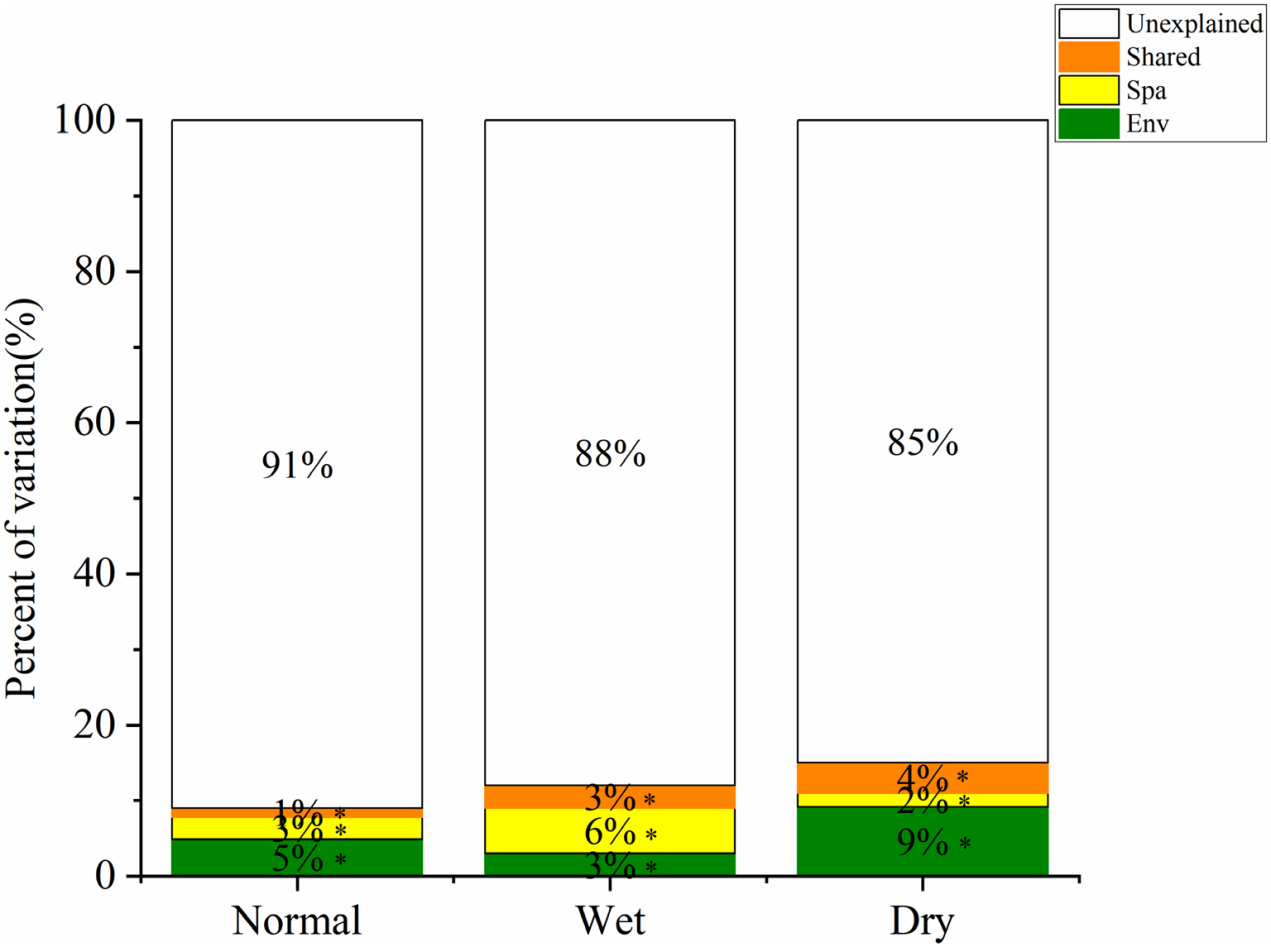
Variation partitioning of the macroinvertebrate metacommunity into pure environmental variables (Env), pure spatial factors (Spa), their shared components (Shared), and unexplained variation across the normal, wet, and dry seasons. The *y*‐axis represents the percentage of variation explained (%), corresponding to the adjusted *R* ^2^ values obtained from variation partitioning using the varpart function in the vegan R package. Environmental and spatial predictors were selected through forward selection using ordiR2step, and significance was assessed via Monte Carlo permutation tests (anova.cca). The figure was created using Origin software based on the VPA results. Asterisks indicate significant components.

The total explained variation in metacommunity structures of river macroinvertebrates ranged from 9% to 15% (Figure 3 and Table S3). Both environmental and spatial factors played important roles in regulating benthic assemblages, although their number, identity, and relative importance varied considerably through time. In the wet season, as expected, the pure effect of spatial factors was obviously more influential than that of environmental variables (6.0% vs. 3.0%). In contrast, during the normal and dry seasons, variations were better explained by environmental variables than by spatial factors (4.9% vs. 2.9% in the normal season; 9.2% vs. 1.8% in the dry season) (Figure 3). The shared effects between environmental and spatial factors also explained a certain percentage of community variation, but were lower than those of pure effects. In addition, substantial unexplained variations were common in the models (Figure 3 and Table S3).

## 4 Discussion

To explore seasonal dynamics in community assembly, we employed an integrated approach that combined the metacommunity structure framework with variation partitioning. We identified distinct seasonal patterns in the idealized metacommunity structures and assessed the relative contributions of deterministic and stochastic processes. Our findings highlight the pivotal role of fluvial hydrological seasonality in driving shifts in community assembly mechanisms: from dispersal‐dominated processes during monsoon‐induced high‐flow periods to environmental filtering mechanisms during normal and drought conditions. The Elements of Metacommunity Structure framework, coupled with variance partitioning analysis, revealed distinct best‐fit patterns across the wet, normal, and dry seasons. These patterns included clumped species loss, quasi‐Clementsian, and Clementsian structures, which align with those frequently observed in previous macroinvertebrate metacommunity studies in lotic ecosystems (Li et al. 2016; Tonkin et al. 2016).

Our ANOVA results revealed significant seasonal variation in key physical parameters, including water temperature, dissolved oxygen, conductivity, water depth, and current velocity (Table 1), reflecting monsoon‐driven hydrological changes. These fluctuations were closely aligned with shifts in metacommunity structure and assembly mechanisms. These seasonal environmental shifts were closely aligned with the observed transitions in metacommunity structures and underlying assembly processes. For instance, during the wet season, elevated water temperature and flow velocity likely enhanced dispersal potential and river connectivity, fostering conditions favorable for mass effects. This corresponds with the nested metacommunity structure identified for the wet season, which often emerges when high dispersal overrides environmental filtering (Liu et al. 2024). Conversely, during the dry season, reduced hydrological connectivity, lower DO levels, and stable but constrained environmental conditions appear to limit dispersal and promote stronger environmental filtering, as indicated by the Quasi‐Clementsian pattern (Liu et al. 2024). During the normal flow period, the intermediate levels of environmental heterogeneity and connectivity may allow both dispersal and filtering processes to act simultaneously, resulting in a Clementsian gradient.

Among these seasonal shifts, the transition from dry to wet periods—driven by monsoon‐induced hydrological changes—plays a particularly critical role in reshaping habitat conditions and macroinvertebrate community assembly dynamics (Leung and Dudgeon 2011; Fernandes et al. 2014). In the Huishui River, monsoon‐driven habitat homogenization likely simplifies community structure while reducing the impact of environmental filtering (Bozelli et al. 2015; Liu et al. 2024). Furthermore, monsoon floods increase spatial connectivity among stream sites (Thomaz et al. 2007), promoting higher dispersal rates via drift (e.g., mass effects). This enhanced dispersal can obscure the effects of environmental filtering on species distributions (Leibold et al. 2004). As a result, strong source‐sink dynamics allow organisms to persist in patches with suboptimal conditions (Leibold et al. 2004; Csercsa et al. 2019). This seasonal shift is also linked to the morphological, phenological, and dispersal characteristics of macroinvertebrates. During the wet season, species such as mayflies, chironomids, and stoneflies dominate the Huishui River. These organisms tend to have small body sizes and short lifespans, typically lasting less than 6 months (Ge et al. 2023). Their dispersal capabilities vary throughout the year (Csercsa et al. 2019), with peak dispersal occurring during the summer metamorphosis from aquatic larvae to winged adults (Morse et al. 1984; Csercsa et al. 2019). Two synergistic mechanisms promote effective dispersal in these taxa: (1) demographic advantages, such as larger population sizes, high fecundity, and rapid generational turnover, which drive frequent dispersal events (Ge et al. 2023); and (2) aerodynamic adaptations that enable passive wind‐assisted transport over long distances. Together, these factors support widespread colonization across the watershed during peak hydrological connectivity (Tonkin et al. 2018). Finally, stochastic processes, such as random dispersal and demographic stochasticity (e.g., birth and death events or ecological drift), may also influence community dynamics (Leibold et al. 2004). Monsoon‐driven floods can lead to stochastic mortality and ecological drift, reducing macroinvertebrate abundance and community size during the wet season. This may contribute to greater spatial heterogeneity among stream sites due to random mortality and colonization events (Liu et al. 2024).

The observed metacommunity structure patterns during the wet season further support the operation of source‐sink (or mass effects) dynamics. As anticipated, we detected a nested pattern in macroinvertebrate communities, characterized by negative range turnover and clustered species loss during this season. In the wet season, certain taxa may be unable to withstand the continuous flooding and drastic alterations in abiotic conditions, leading to their local extirpation. These taxa might then seek refuge in specific areas of the river, where they can persist until conditions stabilize, after which they may recolonize other sites. Meanwhile, taxa that thrive under the altered conditions would proliferate and expand their distributions, a process facilitated by mass effects dynamics (Pulliam Ronald 1988; Liu et al. 2024). This results in a nested metacommunity structure, with narrowly distributed taxa (constrained by specific environmental tolerances) occupying subsets of sites inhabited by more broadly distributed generalists. In summary, mass effects and random recolonization following local extirpations during floods contribute to the dominant role of stochastic spatial processes in shaping community structure during the wet season.

Our findings underscore the dominant role of environmental filtering in shaping macroinvertebrate community assembly during the dry season. This pattern can be attributed to two key ecological mechanisms. First, macroinvertebrates are highly sensitive to environmental stressors, making them effective bioindicators for tracking seasonal hydrological and habitat changes in aquatic ecosystems (Heino et al. 2015; Liu et al. 2024). Second, dry season communities are composed of numerous specialist taxa, particularly bivalves and gastropods, which have distinct environmental niche preferences (Liu et al. 1993). These habitat specialists exhibit divergent microhabitat requirements: bivalves predominantly occupy lentic environments with fine sedimentary substrates (Cao et al. 2012), while semisulcospirid mollusks are strongly associated with lotic habitats containing coarse‐grained sediments (Liu et al. 1993; Du and Yang 2019). Such specialized habitat dependencies restrict their distribution to areas that meet specific environmental conditions. This strong environmental filtering is particularly characteristic of mountain stream ecosystems, which are marked by steep gradients, low temperatures, high habitat heterogeneity, and strong hydrological seasonality. In these systems, macroinvertebrate communities tend to exhibit high levels of ecological specialization and regional endemism, but also limited dispersal capacity and long‐life cycles. These traits make them highly susceptible to habitat alteration and climatic variability (Liu et al. 2024; Li et al. 2025). Therefore, the pronounced niche specificity observed in our study not only reflects seasonal dynamics in community assembly, but also highlights the ecological vulnerability and diagnostic value of macroinvertebrate assemblages in subtropical mountain streams.

The macroinvertebrate metacommunity structure exhibited a Clementsian pattern during the dry season, as evidenced by high coherence, significant species turnover, and positive boundary clumping. This typology—characterized by discrete species assemblages replacing each other along environmental gradients through group‐wise turnover (Clements 1916)—emerges when environmental filtering dominates community assembly processes (Winemiller et al. 2019). This inference is consistent with the results of variation partitioning analysis. The observed patterns likely arose from hydrological fragmentation during seasonal droughts. As water connectivity decreased, previously continuous habitats became isolated patches, reducing organismal dispersal capacity. This fragmentation decoupled local communities from regional species pools, limiting the influence of mass effects and enhancing environmental selection (Dong et al. 2021). As a result, species distributions became strongly linked to specific environmental conditions, following the species sorting paradigm. Discrete community types emerged, with environmental specialists persisting only in suitable habitats, while inferior competitors were excluded through niche‐based filtering mechanisms (Leibold et al. 2004).

The macroinvertebrate metacommunity exhibited a Quasi‐Clementsian structure during the transitional season, a pattern characterized by species turnover that was statistically indistinguishable from random expectations (Tonkin et al. 2016). This intermediate typology suggests a weakened community structuring mechanism compared to the deterministic Clementsian structure observed in the dry season. Specifically, environmental filtering appeared less influential during this period, as shown by variation partitioning results, which indicated similar contributions from both environmental and spatial effects. However, the dispersal rates of organisms during this season were not high enough to generate strong spatial patterns that outweighed the influence of local environmental factors. Consequently, the organization of the metacommunity during the transitional season was more effectively explained by environmental filters than by spatial effects.

This study highlights the role of monsoon‐driven hydrological fluctuations as a critical factor shaping the temporal dynamics of stream macroinvertebrate communities. Our analysis reveals distinct assembly mechanisms across different seasonal hydrological phases: (1) Wet season dominance of stochastic processes—Enhanced hydrological connectivity and reduced habitat heterogeneity favored random community assembly. (2) Dry season shift to deterministic control—Environmental filtering became the predominant force as habitat fragmentation intensified. (3) Transitional equilibrium in the intermediate season—This period between flood regimes was characterized by a balanced contribution from both assembly mechanisms, reflecting the gradual transition in hydrological conditions.

Notably, a large proportion of variation (85%–91%) remained unexplained across seasons, despite the inclusion of environmental and spatial predictors. Such low explanatory power is common in studies using variation partitioning. For example, Li et al. (2023) reported that local, climatic, and spatial factors together explained only 16% of macroinvertebrate variation on the Qinghai–Tibet Plateau, while 84% remained unexplained. Similarly, Chen et al. (2022) found unexplained variation exceeding 80% in seasonal fish communities in Poyang Lake. In mountain stream ecosystems, several factors may account for such low explanatory power. First, stochastic processes, including ecological drift, random colonization, and local extinctions, are likely to play an important role in small, spatially disconnected habitats (García Girón et al. 2021). Second, key environmental drivers—such as microhabitat heterogeneity, substrate structure, or fine‐scale hydrological dynamics—may not be fully captured by the variables used (Tonkin et al. 2017). Third, biotic interactions, such as predation, competition, and life‐history synchrony, are not incorporated in variation partitioning but can significantly influence community structure (Dini‐Andreote et al. 2015; García‐Girón et al. 2020). Additionally, historical effects and temporal mismatches between biological responses and environmental shifts may further obscure patterns (Fukami 2015). As noted by García Girón et al. (2021), variation partitioning cannot account for species interactions or neutral processes, highlighting both methodological limitations and the ecological complexity of community assembly in dynamic headwater systems.

Our findings offer important insights for biodiversity conservation and riverine ecosystem management. Three key implications emerge: (1) Optimizing bioassessment protocols**—**Effective ecological monitoring relies on the dominance of environmental filtering in community organization, as shown by the strong species‐environment correlations during deterministic assembly phases (Liu et al. 2024). During high‐flow periods, seasonal mass effects may decouple biotic responses from environmental conditions, potentially compromising the reliability of assessments (Patrick et al. 2021). (2) Prioritizing habitat management—Conservation strategies should focus on preserving microhabitat heterogeneity to support niche‐based community assembly (Heino and Tolonen 2017). Additionally, maintaining hydrological connectivity is essential for facilitating metacommunity dynamics through organism dispersal, particularly between fragmented habitats (Tonkin et al. 2018; Sarremejane et al. 2021). (3) Adopting a temporal monitoring framework—Given the pronounced seasonal shifts in assembly mechanisms, biodiversity management plans should incorporate regular seasonal sampling to better capture the complexities of ecosystem dynamics (Liu et al. 2024).

## 5 Acknowledge

Although our results revealed clear seasonal patterns in metacommunity structure and quantified the relative contributions of environmental and spatial processes, several limitations must be acknowledged.

First, our analyses did not incorporate direct information on biotic interactions such as predation, competition, or facilitation among species. This limitation stems from the methodological constraints of the variation partitioning and EMS (Elements of Metacommunity Structure) frameworks, which rely primarily on species distributions and abiotic gradients. While the observed patterns of coherence, turnover, and boundary clumping offer indirect evidence of deterministic processes, they may conflate environmental filtering with underlying biotic influences and unmeasured local factors (Leibold and Mikkelson 2002; Henriques‐Silva et al. 2013). To address this, future studies should incorporate more direct evidence of species interactions. For example, experimental manipulations, functional trait‐based analyses, or the development of species interaction networks could provide critical insight into how biotic processes shape community assembly across seasons. Such approaches would significantly improve our ability to disentangle biotic filtering from environmental and stochastic drivers, particularly in dynamic ecosystems such as headwater mountain streams.

Second, while we adopted a seasonal framework to capture the influence of hydrological variation on community assembly, our interpretation remains primarily grounded in an ecological–mechanistic paradigm. However, the contrasting seasonal metacommunity structures and shifting dominance of deterministic versus stochastic processes may also reflect deeper evolutionary and systemic processes. Macroinvertebrates in subtropical mountain streams often exhibit limited dispersal ability, long life cycles, and strong ecological specialization—traits that are likely shaped by long‐term evolutionary adaptation to mountain and monsoonal climatic regimes. These evolutionary constraints may interact with contemporary filtering processes to drive the seasonal patterns we observed (Garcia‐Giron et al. 2019). To move toward a more evolutionary–systemic paradigm, future research should integrate phylogenetic information, trait evolution models, and multiscale interaction networks. This would not only improve mechanistic understanding of metacommunity dynamics but also reveal how evolutionary legacies and systemic feedbacks shape biodiversity in temporally variable environments.

Lastly, a considerable portion of variation in community structure remained unexplained in our models. This residual variation may arise from microhabitat heterogeneity, unmeasured physicochemical variables, or local disturbances that were not captured in our spatial or environmental matrices. Hierarchical modeling frameworks or high‐resolution spatial sampling may be required to improve explanatory power and account for fine‐scale ecological processes.

In summary, advancing our understanding of metacommunity dynamics in complex ecosystems like subtropical mountain streams will require a more integrative approach that combines abiotic drivers, biotic interactions, evolutionary history, and system‐level organization. The present study provides an empirical baseline and conceptual foundation for such efforts.

## Author Contributions

**Yihao Ge:** conceptualization (lead), formal analysis (equal), methodology (equal), writing – original draft (lead). **Yan Xue:** investigation (equal). **Ke Chen:** investigation (equal). **Zhiwen Gan:** formal analysis (equal), methodology (equal). **Ke Xu:** investigation (equal), methodology (equal). **Changrui Zhao:** formal analysis (equal), methodology (equal). **Yuzhou Zhang:** formal analysis (equal), methodology (equal). **Yunzhi Yan:** writing – review and editing (equal).

## Conflicts of Interest

The authors declare no conflicts of interest.

## Supporting information


**Appendix S1:** ece372109‐sup‐0001‐AppendixS1.zip.

## Data Availability

All the required data are uploaded as Supporting Information.
