# Supplementary material for: Seasonal Dynamics of Benthic Macroinvertebrate Community Assembly in a Subtropical Monsoon‐Driven Mountain Stream
Source: Ecol Evol. 2025 Sep 30;15(10):e72109. doi: 10.1002/ece3.72109 (PMC12483986; doi:10.1002/ece3.72109)
Supplement: Supplementary file 1 — Appendix S1: ece372109‐sup‐0001‐AppendixS1.zip. [file ECE3-15-e72109-s001.zip › Appendix.doc]

TabS1 Presence-Absence Data of macroinvertebrate species in Huishui river

|  | Normal | Wet | Dry |
| --- | --- | --- | --- |
| Annelida |  |  |  |
| Oligochaeta |  |  |  |
| Naididae |  |  |  |
| Branchiodrilus sp. | + | + |  |
| Nais sp. | + | + |  |
| Unicinais sp. | + |  |  |
| Pristina sp. | + |  |  |
| Paranais sp. | + |  | + |
| Stylaria fossularis |  | + |  |
| Tubificidae |  |  |  |
| Tubifex sp. | + |  |  |
| Bothrioneurum sp. | + |  |  |
| Limnodrilus sp. | + | + |  |
| Limnodrilus hoffmeisteri | + |  |  |
| Teneridrilus sp. | + |  |  |
| Ilyodrilus sp. | + |  |  |
| Branchiura sowerbyi | + | + | + |
| Hirudinea |  |  |  |
| Glossiphonidae |  |  |  |
| Glossiphonia sp. | + |  | + |
| Piscicolidae |  |  |  |
| Piscicola sp. | + |  | + |
| Erpobdellidae |  |  |  |
| Erpobdella sp. |  |  |  |
| Erpobdella octoculata | + | + | + |
| Salifidae |  |  |  |
| Barbronia sp. |  |  |  |
| Mollusca |  |  |  |
| Gastropoda |  |  |  |
| Viviparidae |  |  |  |
| Bellamya smith |  | + |  |
| Bellamya quadrata | + | + |  |
| Bellamya aeruginosa |  | + |  |
| Bithyniidae |  |  |  |
| Bithynia fuchsiana |  | + |  |
| Bithynia misella |  | + |  |
| Pleuroseridae |  |  |  |
| Semisulcospira libertine | + | + | + |
| Semisulcospira cancellata | + | + | + |
| Assimineidae |  |  |  |
| Assiminca violacea |  | + |  |
| Stenothyridae |  |  |  |
| Stenothyra glabra | + | + |  |
| Physidae |  |  |  |
| Physa acuta |  | + |  |
| Lymnaeidae |  |  |  |
| Lymnaea stagnalis |  |  | + |
| Radix auricularia |  |  | + |
| Radix swinhoei | + | + | + |
| Radix lagotis |  |  | + |
| Radix ovata |  | + |  |
| Radix plicatula |  | + |  |
| Planorbidae |  |  |  |
| Hippeutis umbilicalis |  | + |  |
| Bivalvia |  |  |  |
| Mytilidae |  |  |  |
| Limnoperna fortunei |  | + |  |
| Unionidae |  |  |  |
| Schistodesmus lampreyanus |  |  | + |
| Lamprotula leai |  | + | + |
| Corbiculidae |  |  |  |
| Corbicula fluminea | + | + | + |
| Arthropoda |  |  |  |
| Crustacea |  |  |  |
| Asellidae |  |  |  |
| Asellidae sp. | + |  |  |
| Palaemonidae |  |  |  |
| Macrobrachium sp. |  |  | + |
| Palaemonetes sinensis |  | + |  |
| Atyoidae |  |  |  |
| Caridina sp. | + | + |  |
| Potamidae |  |  |  |
| Potamidae sp. | + | + |  |
| Insecta |  |  |  |
| Ephemeroptera |  |  |  |
| Baetidae |  |  |  |
| Baetiella sp. | + |  |  |
| Centroptilum sp. | + |  | + |
| Cloeon sp. |  | + | + |
| Procloeon sp. |  | + |  |
| Baetis sp. | + | + | + |
| Raptobaetopus sp. |  |  | + |
| Leptophlebiidae |  |  |  |
| Choroterpes sp. | + |  |  |
| Leptophlebia sp. | + |  | + |
| Ephemeridae |  |  |  |
| Ephemera sp. | + | + | + |
| Heptageniidae |  |  |  |
| Heptagenia sp. | + | + | + |
| Cinygmina sp. |  |  | + |
| Epeorus sp. |  |  | + |
| Cinygmula sp. | + |  |  |
| Rhithrogena sp. | + |  |  |
| Ecdyonurus sp. |  | + |  |
| Siphlonuridae |  |  |  |
| Siphlonurus sp. | + | + |  |
| Potamanthidae |  |  |  |
| Potamanthus sp. | + | + | + |
| Ephemerellidae |  |  |  |
| Drunella sp. | + | + |  |
| Ephemerella sp. | + | + | + |
| Isonychiidae |  |  |  |
| Isonychia sp. | + |  |  |
| Caenidae |  |  |  |
| Caenis sp. | + | + | + |
| Brachycercus sp. | + |  |  |
| Plecoptera |  |  |  |
| Perlidae |  |  |  |
| Togoperla sp. |  |  | + |
| etropina sp. |  |  | + |
| Odonata |  |  |  |
| Libellulidae |  |  |  |
| Libellula sp. |  |  | + |
| Macromiidae |  |  |  |
| Macromia sp. | + | + |  |
| Corduliidae |  |  |  |
| Epitheca sp. |  | + |  |
| Cordulegasteridae |  |  |  |
| Cordulegaster sp. | + |  | + |
| Gomphidae |  |  |  |
| Ictinogomphus rapax |  | + |  |
| Lamelligomphus sp. | + | + | + |
| Gomphus sp. | + | + | + |
| Sinictinogomphus sp. | + |  | + |
| Calopterygidae |  |  |  |
| Calopteryx sp. | + | + |  |
| Lestidae |  |  |  |
| Indolestes sp. |  |  | + |
| Trichoptera |  |  |  |
| Hydropsychidae |  |  |  |
| Hydropsyche sp. | + | + | + |
| Ceratopsyche sp. | + | + |  |
| Lepidostomatidae |  |  |  |
| Lepidostomatidae sp. |  | + | + |
| Goeridae |  |  |  |
| Goeridae sp. | + |  | + |
| Philopotamidae |  |  |  |
| Philopotamidae sp. |  |  | + |
| Stenopsychidae |  |  |  |
| Stenopsyche sp. | + | + |  |
| Limnephilidae |  |  |  |
| Limnephilidae sp. | + |  | + |
| Glossosomatidae |  |  |  |
| Glossosoma sp. | + |  | + |
| Rhyacophilidae |  |  |  |
| Rhyacophilidae sp. |  |  | + |
| Rhyacophia sp. |  | + |  |
| Polycentropodidae |  |  |  |
| Polycentropodidae sp. |  |  | + |
| Hydroptilidae |  |  |  |
| Hydroptila sp. |  |  | + |
| Leptoceridae |  |  |  |
| Leptocerus sp. |  | + |  |
| Coleoptera |  |  |  |
| Carabidae |  |  |  |
| Carabidae sp. |  | + |  |
| Dytiscidae |  |  |  |
| Dytiscidae sp1 | + |  | + |
| Dytiscidae sp2 | + |  | + |
| Noteridae |  |  |  |
| Noterus sp. |  |  | + |
| Psephenidae |  |  |  |
| Psephenidae sp. | + | + | + |
| Elmididae |  |  |  |
| Elmididae sp. |  | + |  |
| Stenelmis sp1 | + | + | + |
| Hydrophilidae |  |  |  |
| Hydrophilidae sp. | + |  | + |
| Hemiptera |  |  |  |
| Aphelocheiridae |  |  |  |
| Aphelocheirus sp. |  | + | + |
| Corixidae |  |  |  |
| Sigara sp. | + | + |  |
| Lepidoptera |  |  |  |
| Nymphulinae |  |  |  |
| Elophila sp. |  |  | + |
| Parapoynx sp. |  | + |  |
| Megalogtera |  |  |  |
| Corydalidae |  |  |  |
| Protohermes sp. | + |  | + |
| Neochauliodes sp. |  | + |  |
| Diptera |  |  |  |
| Ceratopogonidae |  |  |  |
| Ceratopogonidae sp. |  | + | + |
| Empididae |  |  |  |
| Empididae lsp. | + | + | + |
| Tabanidae |  |  |  |
| Tabanidae sp. | + | + | + |
| Dolichopodidae |  |  |  |
| Dolichopodidae sp. |  |  | + |
| Rhagionidae |  |  |  |
| Rhagionidae sp. |  |  | + |
| Athericidae |  |  |  |
| Athericidae sp. |  |  | + |
| Tipulidae |  |  |  |
| Tipula sp. | + | + | + |
| Limnophila sp. | + | + | + |
| Hexatoma sp. |  | + | + |
| Blephariceridae |  |  |  |
| Blephariceridae pupa |  |  | + |
| Blephariceridae sp. |  | + |  |
| Simuliidae |  |  |  |
| Simuliidae sp. |  | + | + |
| Simuliidae pupa | + |  |  |
| Simulium sp. | + |  |  |
| Chironomidae |  |  |  |
| Chironomidae pupa | + | + | + |
| Chironomus sp. | + | + | + |
| Cryptochironomus sp. | + | + | + |
| Demicryptochironomus sp. | + |  |  |
| Clinotanypus sp. | + |  |  |
| Einfeldia sp. |  |  | + |
| Dicrotendipes sp. | + |  | + |
| Larsia sp. | + | + | + |
| Rheopelopia sp. | + |  |  |
| Pelopia sp. |  |  | + |
| Rheocricotopus sp. | + |  | + |
| Glyptotendipes sp. | + | + | + |
| Polypedilum sp1 | + | + | + |
| Lipiniella sp. | + |  |  |
| Stictochironomus sp. | + |  | + |
| Tanytarsus sp. | + | + | + |
| Orthocladius sp. | + | + | + |
| Psectrocladius sp. | + | + | + |
| Cricotopus sp. | + | + | + |
| Diamesa sp. | + |  | + |
| Others |  |  |  |
| Nematoda sp. | + | + | + |
| Turbellaria sp. | + | + | + |

TabS2 The average density of characteristic species in different seasons in Huishui River (ind./m^2^) and their percentage contribution to similarity within the group (%)

| Seasons | Normal | Wet | Dry |
| --- | --- | --- | --- |
| *Cryptochironomus* sp. | 117.2（28.0） | 9.6（14.5） | 14.4(7.8) |
| *Stictochironomus* sp. | 61.8（19.8） |  |  |
| Turbellaria sp. | 45.6（18.7） |  |  |
| *Corbicula fluminea* | 36.2（5.4） |  | 33.8(27.8) |
| *Baetis* sp. | 18.6（4.48） | 21.7（13.3） | 56.2(13.4) |
| *Larsia* sp. | 12.8（3.57） |  |  |
| *Radix swinhoei* | 20.6（6.9） | 7.46（8.4） |  |
| *Polypedilum* sp. | 42.8（4.77） | 6.4（7.2） |  |
| *Hydropsyche* sp. |  | 219.2（36.8） |  |
| *Caridina* sp. |  | 12.4（6.2） | 44.3(15.1) |
| *Heptagenia* sp. |  | 11.9（3.37） | 29.6(14.4) |
| *Trissopelopia* sp. |  |  | 16.6(6.4) |
| *Potamanthus* sp. |  |  | 7.6(5.5) |
| total | 355.6（91.6） | 288.7（89.8） | 202.5（90.4） |
| within-group similarity | 42.60% | 40.30% | 50.10% |

**FigureS1 Species distribution patterns of the Hui Shui River in normal, dry and wet seasons**

Table S3a Results of forward selection analysis showing the relationship among β diversity and significant environmental variables in the Huishui River. The table shows the total adjusted R^2^ value of the model after the driver has been selected

| Season | Environmental Variable | Adj.R^2^ | F | P |
| --- | --- | --- | --- | --- |
| Normal | Water depth | 0.061 | 2.296 | 0.010 |
| Wet | Percentage of cobble | 0.060 | 2.274 | 0.012 |
| Dry | Percentage of sand and silt, Land Cover | 0.132 | 2.337 | 0.006 |

Table S3b Results of forward selection analysis showing the relationship among β diversity and significant spatial variables in the Huishui River. The table shows the total adjusted R^2^ value of the model after the driver has been selected

| Season | Spatial Variable | Adj.R^2^ | F | P |
| --- | --- | --- | --- | --- |
| Normal | PCNM2 | 0.041 | 1.847 | 0.050 |
| Wet | PCNM4＋PCNM6 | 0.090 | 1.921 | 0.046 |
| Dry | PCNM11 | 0.058 | 2.229 | 0.010 |
